# Metabolic features of cancer cells in NRF2 addiction status

**DOI:** 10.1007/s12551-020-00659-8

**Published:** 2020-02-28

**Authors:** Keito Okazaki, Thales Papagiannakopoulos, Hozumi Motohashi

**Affiliations:** 1 Department of Gene Expression Regulation, Institute of Development, Aging and Cancer, Tohoku University, Sendai, 980-8575 Japan; 2 Department of Pathology, New York University School of Medicine, 550 First Avenue, New York, NY 10016 USA

**Keywords:** Glutathione synthesis, Glutamate, Non-essential amino acids, Cysteine, Sulfur metabolism, Metabolic liabilities, KEAP1/NRF2

## Abstract

The KEAP1-NRF2 system is a sulfur-employing defense mechanism against oxidative and electrophilic stress. NRF2 is a potent transcription activator for genes mediating sulfur-involving redox reactions, and KEAP1 controls the NRF2 activity in response to the stimuli by utilizing reactivity of sulfur atoms. In many human cancer cells, the KEAP1-mediated regulation of NRF2 activity is abrogated, resulting in the persistent activation of NRF2. Persistently activated NRF2 drives malignant progression of cancers by increasing therapeutic resistance and promoting aggressive tumorigenesis, a state termed as NRF2 addiction. In NRF2-addicted cancer cell, NRF2 contributes to metabolic reprogramming in cooperation with other oncogenic pathways. In particular, NRF2 strongly activates cystine uptake coupled with glutamate excretion and glutathione synthesis, which increases consumption of intracellular glutamate. Decreased availability of glutamate limits anaplerosis of the TCA cycle, resulting in low mitochondrial respiration, and nitrogen source, resulting in the high dependency on exogenous non-essential amino acids. The highly enhanced glutathione synthesis is also likely to alter sulfur metabolism, which can contribute to the maintenance of the mitochondrial membrane potential in normal cells. The potent antioxidant and detoxification capacity supported by abundant production of glutathione is achieved at the expense of central carbon metabolism and requires skewed metabolic flow of sulfur. These metabolic features of NRF2 addiction status provide clues for novel therapeutic strategies to target NRF2-addicted cancer cells.

## KEAP1-NRF2 system as a sulfur-utilizing defense mechanism

**Fig. 1:**
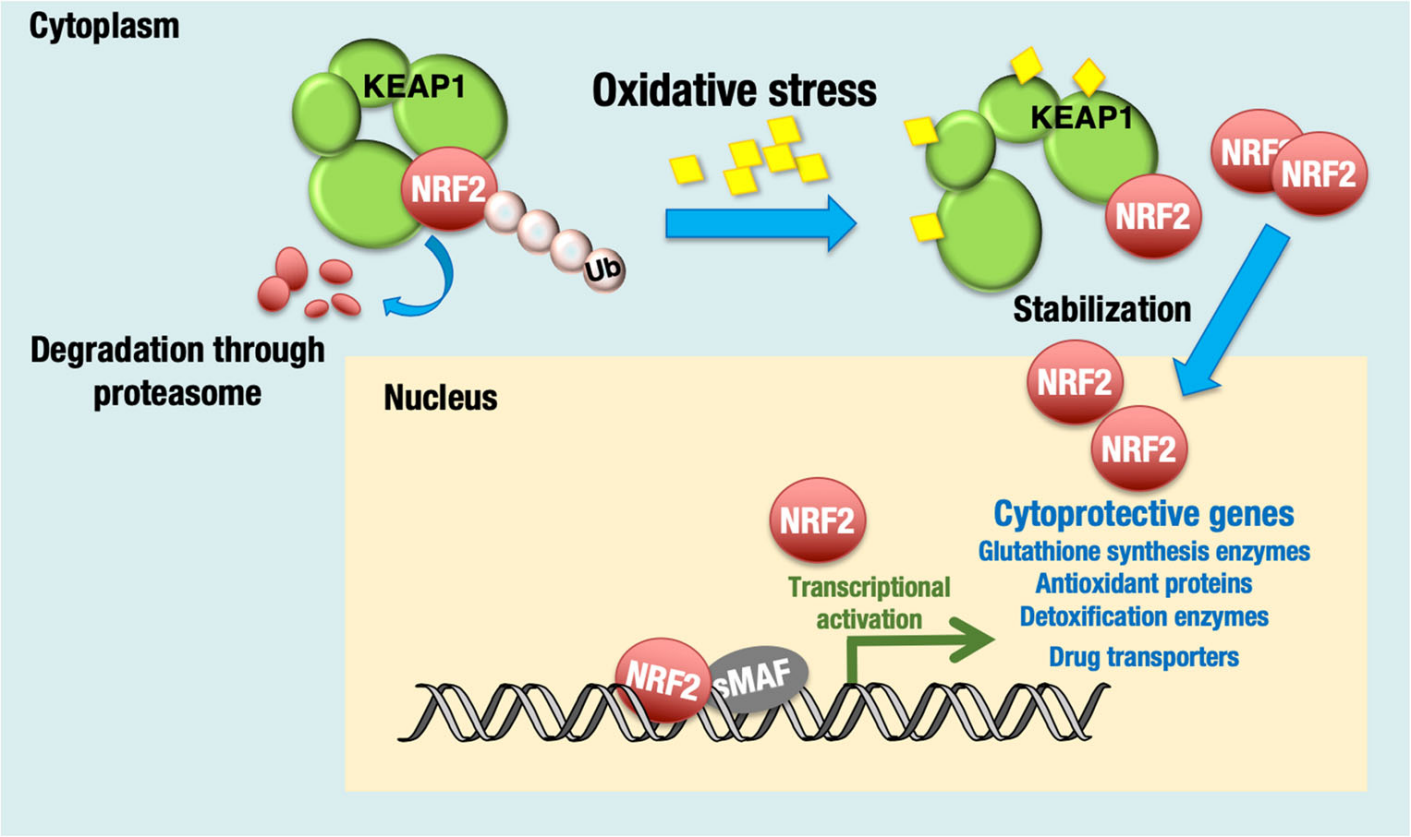
KEAP1-NRF2 system for oxidative stress response. NRF2 is a transcription activator and regulates many cytoprotective genes. In unstressed condition, NRF2 is bound by KEAP1 and ubiquitinated for degradation. In response to oxidative stress, NRF2 is stabilized, translocates into the nucleus, heterodimerizes with small Maf (sMAF), and activates transcription

KEAP1-NRF2 system plays a central role in the defense mechanism from oxidative and electrophilic stresses (Yamamoto et al. 2018) (Fig. 1). NRF2 is a potent transcriptional activator regulating many cytoprotective genes that are involved in detoxification and anti-oxidant function. KEAP1 is a negative regulator of NRF2, serving as a substrate-recognizing subunit of CUL3-based ubiquitin E3 ligase for NRF2 (Yamamoto et al. 2018).

One of the most important features of the KEAP1-NRF2 system is its transient and inducible nature (Yamamoto et al. 2018). Under unstressed condition, NRF2 is constantly ubiquitinated by KEAP1-CUL3 ubiquitin E3 ligase, in which KEAP1 serves as a substrate recognition subunit. When cells are exposed to oxidative and/or electrophilic stress, KEAP1 thiol residues are directly modified, which inactivates the ubiquitin E3 ligase activity of KEAP1-CUL3 complex, leading to stabilization of NRF2. Namely, KEAP1 is a redox biosensor utilizing reactivity of sulfur atoms, switching on the NRF2-mediated transcriptional activation of cytoprotective genes in response to the redox disturbance.

A major output of the NRF2 transcriptional program is involved in redox regulation in which sulfur atoms play a central role, such as glutathione synthesis and reduction, thioredoxin synthesis and reduction, and cystine uptake (Yamamoto et al. 2018). Thus, whereas KEAP1 is a sulfur-utilizing sensor, NRF2 is a sulfur-regulating effector, both together comprising a sulfur-employing defense mechanism.

### NRF2 addiction in cancer cells

In many human cancers, the transient and inducible nature of the KEAP1-NRF2 system is lost. NRF2 is persistently stabilized, resulting in increased amplitude and duration of NRF2 pathway activation. NRF2 pathway activation promotes tumorigenesis by enabling aggressive proliferation and metastasis (Romero et al. 2017; Lignitto et al. 2019) and conferring therapeutic resistance to current standards of care, including chemotherapy and immunotherapy (Jeong et al. 2020; Arbour et al. 2018). These cancer cells are highly dependent on NRF2 activity for their survival and proliferation, a state termed NRF2 addiction (Kitamura et al. 2017; Kitamura and Motohashi 2018).

The aberrant activation of NRF2 is found in various cancers including the lung, gallbladder, esophagus, breast, head and neck, and renal cancers (Shibata et al. 2008; Wang et al. 2010; Shibata et al. 2011; Inoue et al. 2012; Onodera et al. 2014; Kanamori et al. 2015; Martinez et al. 2015; Cancer Genome Atlas Research Network 2016). One of the major causes of aberrant activation of NRF2 in cancer cells is somatic mutation in *KEAP1* or *NRF2* (*NFE2L2*) genes. In particular, *KEAP1* and *NRF2* mutations are encountered in 20–30% of non-small cell lung cancers (Cancer Genome Atlas Research Network 2012; Imielinski et al. 2012; Cancer Genome Atlas Research Network 2014; Campbell et al. 2016). In most cases, they are loss-of-function mutations of *KEAP1* or gain-of-function mutations of *NRF2*, causing the persistent stabilization of NRF2.

Of note, in normal cells, persistent activation of NRF2 due to functional defects of KEAP1-mediated NRF2 degradation is sometimes deleterious, while transient activation of NRF2 in response to various stimuli can be beneficial for our health. Constitutive activation of NRF2 in hematopoietic stem cells in mice promotes proliferation and eventual exhaustion (Murakami et al. 2017) and shortens lifespan of drosophila (Tsakiri et al. 2019). It seems that persistent NRF2 activation increases anti-oxidant and detoxification capacities at the expense of juvenescence. Moreover, from a view point of cell competition, persistent activation of NRF2 primes cells for their elimination by wild-type neighbors, conferring the loser status (Kucinski et al. 2017). These results suggest that NRF2 addiction in cancer cells is established only after overcoming disadvantages and liabilities accompanying the persistent transcriptional activation mediated by NRF2.

“NRF2 addiction” of many cancer cells highlights that NRF2 itself or its target genes should be the most effective therapeutic target from a cell-autonomous point of view. NRF2 is very pleiotropic and there is redundancy in its transcriptional output, therefore targeting of specific NRF2 targets has been therapeutically ineffective. Furthermore, like most transcription factors, direct targeting of NRF2 itself has been extremely challenging. However, if an NRF2 inhibitor existed, administration of such an inhibitors to cancer-bearing patients may not be so advantageous considering the important role of NRF2 in normal cells for stress response and cytoprotection. For example, NRF2 inhibition in cancer-bearing hosts, especially in myeloid cell lineage, promotes metastatic colonization of cancer cells in the lung by augmenting the activity of myeloid derived suppressor cells (Satoh et al. 2010; Hiramoto et al. 2014). On the other hand, oxidative stress in regulatory *T* cells strengthen their suppressor activity, and administration of an NRF2-inducing reagent sulforaphane inhibits tumor growth in allograft experiment (Maj et al. 2017), suggesting that NRF2 activation in cancer-bearing hosts is beneficial. Therefore, alternative therapeutic targets other than NRF2 itself are required for controlling NRF2-addicted cancer cells.

## Trans-omics approach for understanding molecular basis of NRF2-driven cancer malignancy

**Fig. 2:**
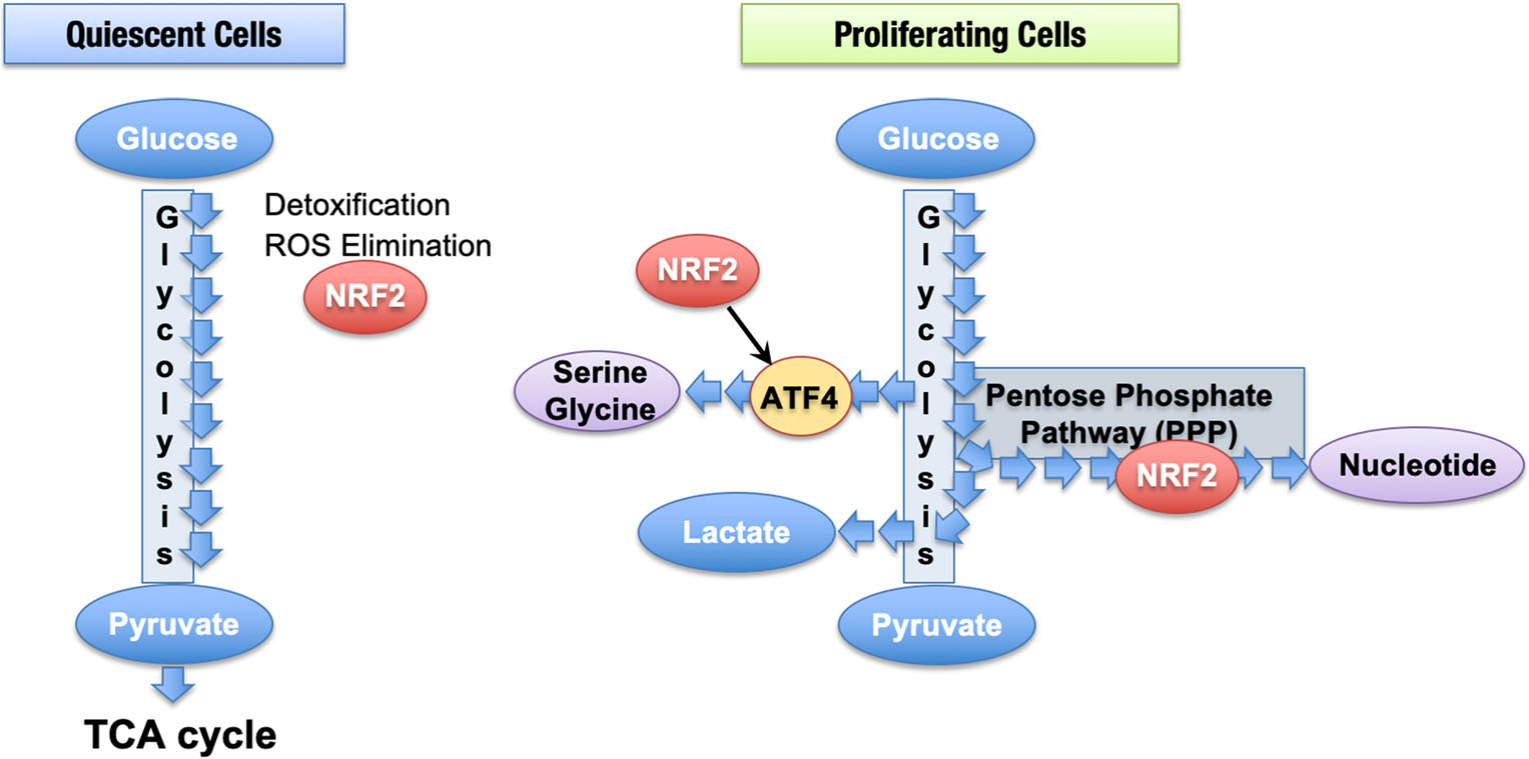
Contribution of NRF2 to metabolism in proliferating cells. Whereas NRF2 mainly contributes to antioxidant function and detoxification in normal quiescent cells, NRF2 contributes to metabolic reprogramming in proliferating cells, facilitating pentose phosphate pathway, and serine synthesis.

Several studies have demonstrated that NRF2 addiction of cancer cells is supported by unique metabolic activities (Fig. 2) (Mitsuishi et al. 2012; DeNicola et al. 2015; Romero et al. 2017). Mitsuishi et al. examined NRF2-dependent transcriptome and NRF2 cistrome (genome-wide NRF2 binding sites) using one of the *KEAP1*-mutant non-small cell lung cancer (NSCLC) cell lines, A549 cells, and found that several metabolic genes, those involved in the pentose phosphate pathway and NADPH production as well as glutathione synthesis, are directly regulated by NRF2. The NRF2-dependent transcriptome data and NRF2 antibody ChIP-seq data were combined with NRF2-dependent metabolome data to verify the functional contribution of the NRF2 target genes to the metabolic regulation. ^13^C-labeled glucose and glutamine were used to trace the destiny of the labeled carbons, which was an effective strategy to show that NRF2 activation skewed the metabolite flow in the cells. Enhancement of purine nucleotide synthesis via the pentose phosphate pathway was found to be advantageous for cell proliferation and tumorigenesis of NRF2-addicted cancer cells (Mitsuishi et al. 2012).

DeNicola et al. examined serine/glycine synthesis activity of multiple NSCLC cell lines by flux analysis using ^13^C-labeled glucose, which were then compared with transcriptome data of these cell lines. The serine/glycine synthesis activities were found positively correlated with expression levels of NRF2 target genes. Although NRF2 does not directly regulate serine synthesizing enzyme genes, ATF4, a key regulator of serine synthesis, was shown to be activated by NRF2. This comprehensive study revealed that serine synthesis branching from the glycolytic intermediates 3-phosphoglycerate is enhanced in NSCLC cells with NRF2 activation (DeNicola et al. 2015).

While multiple advantageous aspects of the metabolic reprogramming for NRF2-addicted cancer cells have been described, metabolic liabilities resulting from NRF2-dependent reprogramming have also been investigated. Romero et al. conducted a CRISPR/Cas9 genetic screen with *KEAP1*-mutant lung cancer cells and found that NRF2-addicted cancer cells are highly dependent on glutamine uptake, and that, consequently, inhibition of glutamine-derived glutamate by glutaminase inhibition effectively suppresses NRF2-addicted lung cancers (Romero et al. 2017; Mukhopadhyay et al. 2020). Satisfying the increased demand for glutamine/glutamate is likely to be one of the critical requirements for the establishment of NRF2-addiction in cancer cells.

### Potent antioxidant function at the expense of central carbon metabolism

**Fig. 3:**
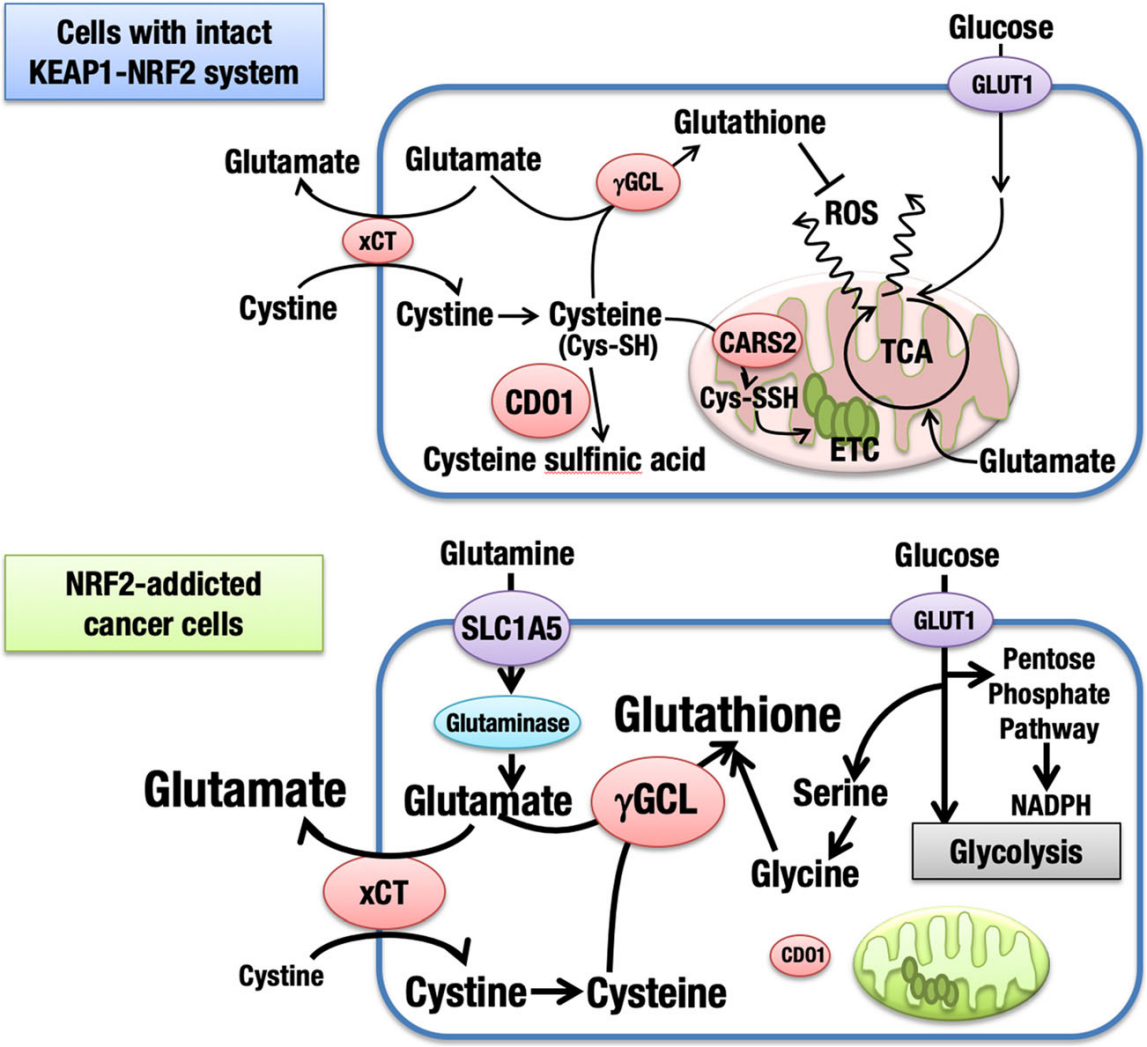
Unique metabolic signature of NRF2-addicted cancer cells. Cells with intact KEAP1-NRF2 system, regardless of normal cells and cancer cells, transiently activated NRF2 promotes glutathione synthesis. Glucose and glutamine are catabolized in mitochondria and fuel TCA cycle. Cysteine is converted to cysteine persulfide (Cys-SSH) by CARS2 and also contributes to the maintenance of the mitochondrial membrane potential. Cysteine is also converted to cysteine sulfinic acid by CDO1. Glutathione is effective to quench reactive oxygen species (ROS) generated from mitochondria. In contrast, NRF2-addicted cancer cells exhibit remarkable enhancement of cystine uptake via xCT and glutathione synthesis, which is coupled with enhanced glutamine uptake to supplement glutamate and CDO1 suppression to further increase the cysteine availability. Glucose-derived serine synthesis also supports glutathione synthesis. Mitochondrial respiration is limited due to redirection of metabolites, which instead flow into glutathione synthesis and other anabolic pathways

The dependency of NRF2-addicted cancer cells on exogenous glutamine is attributable to the elevated consumption and excretion of glutamate as a result of two major transcriptional outputs of NRF2 (Fig. 3, lower panel). One is glutamate excretion coupled with cystine uptake through xCT, an antiporter of cystine and glutamate. The other is glutamate incorporation into glutathione catalyzed by gamma-glutamylcysteine ligase (γGCL), a rate limiting enzyme of glutathione synthesis (Sayin et al. 2017). Indeed, combination of exome, transcriptome, and metabolome analyses of multiple NSCLC cell lines also clearly demonstrated that remarkable enhancement of glutamate excretion, cystine uptake, and glutathione synthesis is a striking and reproducible feature of NRF2-addicted cancer cells (Saigusa et al. 2019).

In cells with intact KEAP1-NRF2 system, transiently stabilized NRF2 induces transcriptional activation of genes encoding xCT, *SLC7A11*, and two subunits of gamma-glutamylcysteine ligase (γGCL), *GCLC* and *GCLM*, in response to oxidative and electrophilic stresses. Because the induction of these genes is only transient, the glutamate consumption is likely to be buffered by steady-state central carbon metabolism, under which glucose and glutamate fuel mitochondria to support cellular bioenergetic demands (Fig. 3, upper panel). In contrast, persistently stabilized NRF2 in response to KEAP1 or NRF2 mutations causes sustained activation of these genes, leading to the accumulation of xCT/SLC7A11, GCLC, and GCLM that cause a depletion of intracellular glutamate (Sayin et al. 2017). In addition, NRF2-addicted cells exhibit further elevated expression of *SLC7A11*, *GCLC,* and *GCLM* due to additional cooperative input to the NRF2-mediated transcriptional activation. Namely, sustained activation of PI3K-AKT pathway dramatically promotes the accumulation of NRF2 by inhibiting KEAP1-independent degradation mechanism of NRF2, resulting in the augmentation of NRF2-mediated transcriptional activation (Mitsuishi et al. 2012; Taguchi et al. 2014). This is because the sustained activation of PI3K-AKT pathway inhibits GSK3, which phosphorylates NRF2 and allows its recognition and ubiquitination by βTrCP-CUL1 ubiquitin E3 ligase for degradation. As for another cooperative factor, ATF4 activation has a synergistic effect with NRF2 activation on xCT expression (Ye et al. 2014; Mimura et al. 2019). Consequently, cystine uptake coupled with glutamate excretion and glutathione synthesis are enhanced (Fig. 3, lower panel).

In order to maintain intracellular glutamine-derived glutamate levels, NRF2-addicted cells exploit glutamine transporter, SLC1A5, to uptake more extracellular glutamine that is then converted to glutamate by glutaminase (Romero et al. 2017). NRF2 redirects glutamate to glutathione synthesis and cystine uptake and away from TCA cycle anaplerosis, therefore limiting glutamate as a carbon source for TCA cycle and mitochondrial activity (Sayin et al. 2017). Additionally, the enhancement of serine synthesis pathway in NRF2-addicted cancer cells (DeNicola et al. 2015) can promote glycine availability for glutathione synthesis (Yang and Vousden 2016; Rodriguez et al. 2019). Thus, NRF2-addicted cancers adopt highly specialized metabolism favoring glutathione synthesis that is essential for the potent anti-oxidant and detoxification capacities, which cannot be achieved by a simple activation of NRF2.

### Cysteine catabolism for mitochondrial energy production

A recent study revealed that mitochondrial sulfur metabolism makes an important contribution to the cellular energy production (Akaike et al. 2017). A mitochondrial enzyme CARS2 generates cysteine persulfide from cysteine, which is regarded as one of the major processes for production of persulfides, i.e., molecular species containing more than one sulfur atoms in thiol moiety. Persulfides possess dual reactivities as electrophiles and nucleophiles (Fletcher and Robson 1963; Parker and Kharasch 1959; Abdolrasulnia and Wood 1980). This unique chemical property makes persulfides favorable substrates for the energy production that basically relies on multiple steps of redox reactions. Indeed, CARS2 inhibition in KEAP1-NRF2-intact cells decreased the mitochondrial membrane potential and oxygen consumption rate (Akaike et al. 2017).

CARS2 is originally known as a mitochondrial isoform of cysteinyl t-RNA synthetase, and the persulfide synthesizing activity is a moon-lighting function of CARS2. The cysteinyl t-RNA synthetases of various species possess four highly conserved motifs. Two of them are critical for the cysteinyl t-RNA synthesis and subsequent protein translation, and the other two are critical for the persulfide synthesis. CARS2 mutant lacking the persulfide synthesis activity did not rescue the decreased mitochondrial membrane potential of CASR2-deficient cells, whereas CARS2 mutant lacking cysteinyl t-RNA synthesis activity did. Thus, mitochondrial persulfide production is regarded necessary for the mitochondrial energy metabolism (Akaike et al. 2017).

The sulfur atoms in persulfides participate in the energy metabolism in mitochondria serving as electron donors as well as electron acceptors and are mostly likely to be excreted as thiosulfate eventually. While the cysteine decomposition is coupled with mitochondrial bioenergetics in the cells with intact KEAP1-NRF2 system, in NRF2-addicted cancer cells, most of the available cysteine appears to flow into the anabolic pathway, namely, glutathione synthesis and leaves the catabolic pathway in mitochondria, which is consistent with the observation that mitochondrial respiration is limited in NRF2-addicted cancer cells (Sayin et al. 2017). Moreover, an alternative pathway of cysteine catabolism, which is mediated by CDO1, has been shown to be inactive in NRF2-addicted cancer cells due to DNA methylation at the *CDO1* locus (Kang et al. 2019). CDO1 converts cysteine to cysteine sulfinic acid, resulting in the limited availability of cysteine for the anabolic pathway. Thus, by silencing CDO1, NRF2-addicted cancer cells maintain a large intracellular pool of cysteine for the glutathione synthesis, resulting from the increased cysteine uptake by xCT and decreased cysteine decomposition.

## Future therapeutic strategy for controlling NRF2-addicted cancers based on their metabolic features

The dependency on glutamine and glutamate is regarded as an Achilles’ heel of NRF2-addicted cancer cells. Therefore, targeting glutaminolysis using glutaminase inhibitors presents a promising therapeutic approach for aggressive subtypes of lung cancer with mutations in KEAP1/NRF2 (Romero et al. 2017). Another interesting approach emerges by focusing on an important role of glutamine and glutamate in the synthesis of non-essential amino acids (NEAAs) (LeBoeuf et al. 2019). Although NEAAs can be synthesized by cells, many cancer cells tend to be dependent on the exogenous supply of NEAAs due to their highly proliferative nature and increased demand of amino acids (Tsun and Possemato 2015). Given that glutamate is a critical nitrogen donor for transamination reactions that generate most NEAAs, limited availability of glutamate in NRF2-addicted cancer cells results in increased dependency on exogenous NEAAs (LeBoeuf et al. 2019). Therefore, restriction of NEAAs, such as serine, glycine, and asparagine, in the tumor microenvironment can effectively inhibit tumorigenesis of NRF2-addicted cancer cells.

To overcome the difficulties in targeting NRF2 for controlling NRF2-addicted cancers, targeting metabolic liabilities unique to NRF2-addicted cancer cells, such as dependency on exogenous NEAAs, including glutamine, is a very sensible way to achieve selective toxicity toward cancers that have fallen in NRF2 addiction. Importantly, persistent activation of NRF2 that is induced pharmacologically in KEAP1-NRF2-intact cancer cells also decreases NEAA synthesis and makes them rely on exogenous NEAAs (LeBoeuf et al. 2019). Administration of NRF2 activator, which would be favorable for reinvigorating anti-tumor immunity and conferring the metabolic liabilities on cancer cells, in combination with inhibitors of glutamate and/or NEAA synthesis may be an effective anti-tumor therapy from the viewpoint of interaction between cancer cells and their microenvironment.

NRF2-addicted cancer cells are very well adapted to the greatly enhanced generation of antioxidant and detoxification capacity by establishing unique metabolism. With abundant synthesis of glutathione and NADPH, a redox balance in NRF2-addicted cancer cells is likely to be shifted toward the reducing condition. An excessive shift in the balance would generate reductive stress leading to the protein aggregates and proteotoxicity as was reported in myocardium (Rajasekaran et al. 2011; Kannan et al. 2013). Further enhancement of the reducing condition might be an alternative approach for the selective toxicity to NRF2-addicted cancer cells by provoking the reductive stress.
